# Effect of urbanization on zoonotic gastrointestinal parasite prevalence in endemic toque macaque (*Macaca sinica*) from different climatic zones in Sri Lanka

**DOI:** 10.1016/j.ijppaw.2021.12.007

**Published:** 2021-12-27

**Authors:** Shehani U. Fernando, PreethiV. Udagama, Saminda P. Fernando

**Affiliations:** a Department of Zoology and Environment Sciences, Faculty of Science, University of Colombo, Colombo 03, Sri Lanka; b Department of Zoology, Faculty of Natural Sciences, The Open University of Sri Lanka, Nawala, Nugegoda, Sri Lanka

**Keywords:** Sri Lanka, Toque macaque, *Macaca sinica*, Gastrointestinal (GI) parasites, Zoonoses, Conservation

## Abstract

Understanding variations in host-parasite relationships with urbanization is vital for both, public health management and conservation of endemic animals with high anthropogenic interactions. Toque macaques (*Macaca sinica*) are such endemic old-world monkeys in Sri Lanka. Three macaque sub species inhabit the main climatic zones of the island; *M.s. sinica,M.s.aurifrons* and *M.s.opisthomelas* inhabit the dry zone, wet zone, and montane regions of the island, respectively. This study aimed to examine parasite prevalence in this host in association with urbanization. A total of 180 fecal samples were collected from the three sub species of toque macaques inhabiting the main climatic zones (dry, wet, and montane) in Sri Lanka; 20 samples each were collected from urban, suburban, and wild populations representing each climatic zone. Twenty gastrointestinal (GI) parasite genera types *i.e*. five types of protozoan cysts, two types of trematode ova, four types of cestode ova, eight types of nematode ova, and a single type of acanthocephalan ova were identified. The overall prevalence of parasites was 62% (112/180) with the highest prevalence of *Strongyloides* infection. In all three sub species, toque macaque populations with proximity to human settlements, including urban and suburban populations, manifested a greater GI parasitic prevalence, mean ova/cyst counts and species richness, compared to their wild counterparts. Importantly, records of five parasite types in toques in Sri Lanka are reported for the first time, while *Moniliformis* type was reported as a first record in free ranging macaques, globally. This study clearly demonstrated that human contact and habitat modification may influence patterns of parasitic infections in macaques. As most of the parasite types identified manifest zoonotic potential, with public health implications, close associations of macaques may cause a threat to human well-being.

## Highlights

• GI parasites of the 3 endemic, endangered Sri Lankan macaque subspecies recorded.
• Parasitic infections were examined in realtion to urbanization of macaque habitats.
• 20 genera types of protozoan, helminth and acanthocephalan groups identified.
• Urban/sub-urban habitats led to significantly higher parasite prevalence than in the wild.
• Moniliformis sp. in macaques was recorded for the first time, globally, along with five novel local records of parasite-toque macaque associations.

## Graphical abstract

**Figure:**
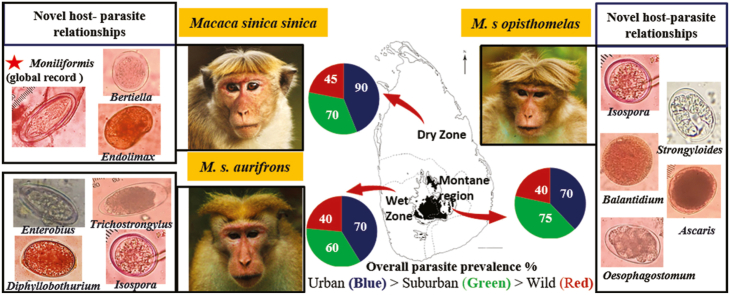

## 1 Introduction

During the past few decades, rapid intensification, and fragmentation of ecosystems through urbanization, were responsible for the rapid and comprehensive change in parasites of primates (Moll et al., 2019). Such modifications and alterations in natural habitats impact upon primate survivability, and susceptibility to parasitic infection, as parasites can influence the health (Johnson and Hoverman, 2012), population size (Tompkins et al., 2002), reproductive condition (Auld et al., 2016), behaviour and social interactions (Herbison et al., 2018) of primates. Due to the loss and fragmentation of their natural habitats, primates are forced to live in anthropogenically disturbed urban and suburban areas with proximity to humans. Thus, continuous human disturbances and encroachment, the ability of some parasites to infect multiple host species, and high level of genetic homology between humans and primates, increase the emergence and transmission of parasitic diseases from primates to humans and *vice versa* (Jones-Engel et al., 2004; Lane et al., 2011; Amarasinghe and Premathilake, 2014). Such interactions provide cause for serious concern on primate conservation as well as on public health. Thus, it is important to emphasize on the effects of human induced landscape changes on GI parasite infection in primates.

Several published studies had focused on parasites on free-ranging, semi-captive and captive orangutans, *M. fascicularis*, and *M. nemestrina* (Mul et al., 2007; Adrus et al., 2018) as well as on several semi-captive and wild primate species (Warren, 2001; Milozzi et al., 2012). Although anthropogenic activities are likely to play a role in primate-parasite associations, none of the previous studies had examined the variations in parasitic infections in the same primate species that differed in their contacts and interactions with humans. Hence, to widen the scope of such investigations, this study specifically focused on the effects of human induced landscape changes on GI parasite infection in toque macaques.

**Fig. 1:**
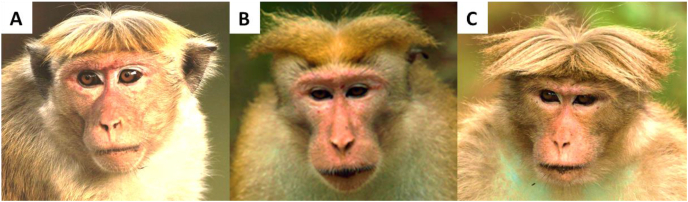
The three subspecies of macaque's endemic to Sri Lanka. (A) Common macaque (*Macaca sinica sinica*), (B) dusky or pale-fronted macaque (*M*. *s*. *aurifrons*), and (C) hill-zone macaque (*M*. *s*. *opisthomelas*) (image courtesy: Madura De Silva).

Toque macaque (*Macaca sinica)* in Sri Lanka is a perfect model for assessing the influence of the anthropogenic landscape on GI parasite infections. They are one of the three primate species endemic to Sri Lanka, the others being purple-faced langur (*Trachypithecus vetulus*) and red slender loris (*Loris tardigradus*) (Huffman et al., 2013). Toque macaques are found as three geographically segregated subspecies across the six climatic zones in the country: the dry zone macaque or common macaque (*M*. *s. sinica,* Linnaeus, 1771) in the dry zone lowland, arid lowlands and intermediate zones; dusky or pale-fronted macaque (*M*. *s*. *aurifrons,* Pocock, 1931) in the rainforests of the lowland and midland of the wet zone, and the hill-zone macaque (*M*. *s*. *opisthomelas,* Hill, 1942) in the montane regions (Dittus, 1977; Nekaris and de Silva Wijeyeratne, 2009; Dittus, 2013) (Fig. 1).

Their populations declined gradually over the past 40 years, where the total population size reduced in half, largely due to habitat loss and persecution of *M*. *sinica* by humans (Nahallage et al., 2008; Nahallage and Huffman, 2013). Therefore, according to the IUCN Red List of Threatened Species, *M. s. sinica* and *M. s. aurifrons* are categorized as “Endangered” (Dittus, 2020; Dittus and Nekaris, 2020) whereas *M. s. opisthomelas* is listed as “Critically Endangered” (Dittus and Gamage, 2020). In addition, macaques are also listed in Appendix II of CITES (Convention on International Trade in Endangered Species) that includes species not necessarily threatened with extinction (CITES, 2005). Even though there are reports on the health, behaviour, and ecology of toque macaques (Dittus, 1984, 1986; Dittus and Ratnayeke, 1989; Keane et al., 1997; Weerasekara et al., 2021), parasite diversity of these animals is not well studied. A single study was reported on the macaque populations across the country, representing the three subspecies (Huffman et al., 2013) while others were mainly focused on macaques inhabiting specific regions, *i.e* Polonnaruwa (*M. s. sinica*), Peradeniya (*M. s. aurifrons*) (Dewit et al., 1991; Ekanayake et al., 2004, 2006; Thilakarathne et al., 2021), and in captivity (Gunasekera et al., 2012; Aviruppola et al., 2016). Hence, this study was undertaken to fulfill two objectives; Firstly, to determine how human influenced landscape modifications facilitated the transmission of GI parasites in macaques (in correlation to parasite prevalence, species-richness, and fecal ova/oocyst/cyst counts) in urban, suburban, and wild populations. Secondly, to establish baseline data for parasites of zoonotic potential of macaques, with a view to project significant impacts on the conservation of endangered macaques, and on the well-being of associated human populations.

## 2 Materials and methods

### 2.1 Ethics statement

This study was conducted with permission from the Department of Wildlife Conservation, Sri Lanka (WL/3/2/32/19) to enter National Parks in Sri Lanka, and to collect macaque fecal samples from these locations. The study was based on noninvasive sample collection following defecation, without causing disturbance or distress to the animals; hence, obtaining ethical clearance was not applicable.

### 2.2 Study sites

This study included the three endemic sub species of toque macaques, *Macaca sinica sinica*, *M*. *s*. *aurifrons*, and *M*. *s*. *opisthomelas,* inhabiting three different climatic zones, the dry zone, wet zone, and the montane region, of the island. Fourteen study sites were selected representing the dry and wet zones and montane regions in the island. The selection was based on earlier records on the presence of subspecies of toque macaques, and the ease of travel to these locations.

To assess the impact of urbanization on parasitism, each subspecies was again divided into three populations based on their level of human contact; urban/free ranging areas with the continuous industrialized expansions, where human contact was higher due to higher human density; suburban, areas adjacent to forests and with less frequent human contact, populated with a low level of human density (lesser than in urban areas); wild macaques that lived in their undisturbed natural habitats, sans involvement with humans. All these locations are geographically segregated and differed significantly in macaque habitats, the degree of anthropogenic disturbances, and climatic conditions.

Dry Zone (*M*. *s*. *sinica*): The dry zone area occupies approximately two-thirds of Sri Lanka's land area (southeast, east, and northern parts), which consists of variable forest structures and different types of habitats. Climatically, the annual rainfall varies between 1250 and 1800 mm and the temperature ranges between 29 and 32 °C. Five study sites in the dry zone were selected based on their level of human contact: Anuradhapura (8°18′46.12"N 80°24′11.38"E), Kurunegala (7°29′0.66"N 80°22′5.69"E) (urban/free ranging); Anuradhapura archeological sites (8°22′10.67"N 80°23′39.98"E), Dambulla (7°51′20.37"N 80°38′59.91"E) (suburban); and Udawalawa National park(6°28′23.91"N 80°53′55.36"E) (wild).

**Fig. 2:**
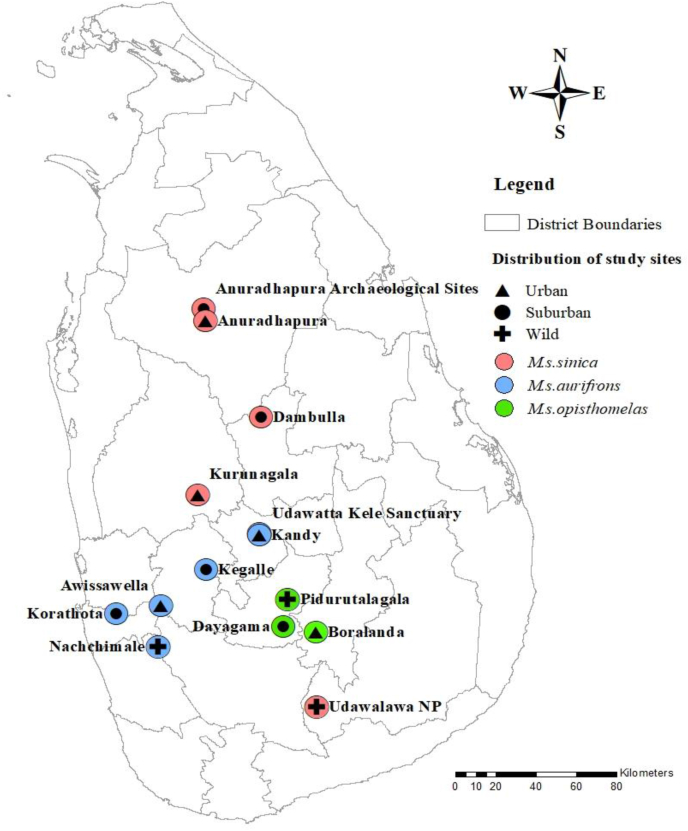
Map of Sri Lanka with sampling localities in the dry and the wet zones and the montane region.

Wet zone (*M*. *s*. *aurifrons*): The southwestern part and the mountains of the island, well known as the “wet zone”, receives an ample annual average rainfall of 2000–2500 mm with temperature that range from 24 to 27 °C. Six geographic locations with *M. s. aurifrons* macaque populations were selected encompassing urban, suburban, and wild habitats (Fig. 2). Samples of the urban/free ranging population were obtained from two locations, Awissawella (6°57′14.41"N 80°12′16.15"E) and Kandy (7°17′35.74"N 80°38′29.36"E), while Korathota (6°54′54.14"N 80° '7.69"E) and Kegalle (7° 7′36.80"N 80°24′13.01"E) harboured suburban macaque populations. Macaque populations inhabiting Udawattakele sanctuary (7°17′55.90"N 80°38′34.02"E) and Nachchimale forest (6°45′31.70"N 80°11′22.52"E) were sampled as wild populations (Fig. 2).

Montane region (*M*. *s*. *opisthomelas*): The montane region located above 1000 m in the central highlands of Sri Lanka, receives 2500–5000 mm annual rainfall with the mean daily temperature being approximately 16 °C. Three sampling sites were selected according to the level of anthropogenic disturbances, including Boralanda (6°49′39.84"N 80°53′38.56"E) (Urban/free ranging), Dayagama (6°51′17.07"N 80°44′53.74"E) (suburban), and Pidurutalagala (6°59′10.25"N 80°46′13.32"E) (wild) (Fig. 2).

### 2.3 Sampling

A total of 180 macaque fecal samples were collected form all three subspecies of macaques, between January and November 2019: 20 samples each from urban, suburban, and wild locations amounting to 60 samples per subspecies. The activity patterns of free-ranging macaques were monitored daily from 8.00 a.m. to 4.00 p.m. Fecal samples of macaque troops were opportunistically collected by following the macaque troop till defecation took place. Upon observing that a macaque had defecated and before collection, the sample was examined for the presence of mucus, blood, and tapeworm proglottids. Then, with gloved hands and a disposable spoon, a sample from within the fecal mass was collected into a plastic tube. The Parafilm sealed tube was labeled with the date, time, location and habitat type, and samples were transported at +8 °C (in a cooler box with ice packs) to the laboratory and stored at 4 °C for a maximum of 96 h, prior to microscopic examination.

### 2.4 Coprological examination

Samples were analyzed at the Zoology Laboratory of the Department of Zoology and Environment Sciences of the Faculty of Science, University of Colombo. Fecal analysis was carried out essentially according to Jenkins et al. (2017), using modified Sheather's sucrose flotation method, suitable for detection and identification of various parasite ova and cysts. Briefly, 3 g of feces was mixed with distilled water and centrifuged thrice (at 2045*×g*). The resultant pellet was emulsified and thoroughly suspended in a saturated sucrose solution. Approximately, 5 ml of the top suspensions were collected, topped up with distilled water and repeatedly centrifuged at 1370×*g* for 10 min. Finally, 1 ml of each suspension with the pellet was transferred to the Eppendorf® tubes. Distilled water was added to a final volume of 1.5 ml then centrifuged for 10 min at 1150×*g.* The supernatants were discarded. The remaining 0.5 ml pellet thus obtained, was observed under standard light microscopy (Olympus, Japan) for the presence of parasite ova and cysts. Parasite stages were identified using available keys and previous references by morphological characteristics (shape, colour and content) and morphometric characteristics (length and width) (Greiner, 1989; WHO, 1994; Kouassi et al., 2015; Klaus et al., 2017; Li et al., 2017).

### 2.5 Parasite indices and statistical analysis

Parasite prevalence was calculated as a proportion of the number of infected individuals to the total number of examined hosts (Hodder and Chapman, 2012). Parasite richness was described as the total of parasite taxonomic groups found through the morphological identification, in each habitat type, of a given macaque subspecies (Gillespie et al., 2005). The total number of ova per gram (OPG) and cysts per gram (CPG) feces for each parasite type was considered as the parasite load (Hodder and Chapman, 2012).

Normality of distributions was tested by the Shapiro-Wilk normality test. Chi-square test determined differences in the overall parasite prevalence of each sub species, habitat type, the relative prevalence of each parasite type and prevalence of multiple infection between urbanized (urban and suburban) and wild habitats. Kruskal-Wallis test assessed differences in parasite richness and parasite ova per gram (OPG) or cysts per gram (CPG) feces, among the three habitat types within a climatic zone. Mann-Whitney *U* test to compare the differences in parasite richness and intensity, in urbanized habitats (urban and sub urban) and wild habitat. All statistical tests were performed at a significance level of *p* < 0.05, using SPSS Statistics V23.0 (IBM, USA). It was assumed that the habitat types the macaques were found during fecal sample collection to be consistent with parasitic infections at that time (Hodder and Chapman, 2012). However, due to the ranging behaviour of macaques, their distribution may have extended beyond a single habitat type.

## 3 Results

### 3.1 GI parasites of macaques

**Fig. 3:**
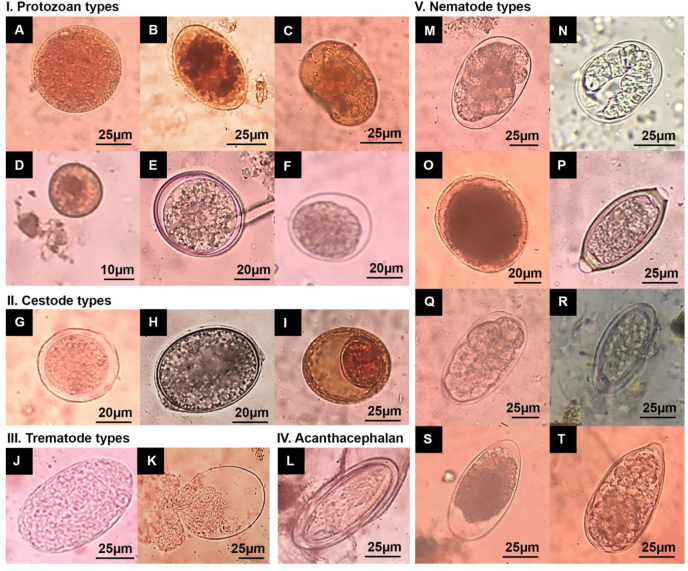
GI parasite genera types identified from fecal samples of toque macaques. I. Protozoan types: (A) *Balantidium* cyst, (B) *Balantidium* trophozoite, (C) *Endolimax* cyst, (D) *Entamoeba* cyst, (E) *Isospora* cyst. (F) Unidentified protozoan cyst; II. Cestode types: (G) *Bertiella* ova, (H) *Diphyllobothrium* ova, (I) *Hymenolepis* ova; III. Trematode types: (J–K) Unidentified trematode ova; IV. Acanthocephalan type: (L) *Moniliformis* ova; V. Nematode types: (M) *Oesophagostomum* ova, (N) *Strongyloides* ova, (O) *Ascaris* ova, (P) *Trichuris* ova, (Q) *Strongyle*/Hookworm ova, (R) *Enterobius* ova, (S)*Trichostrongylus* ova, (T) Unidentified nematode ova.

The three main parasite groups identified in this study were protozoans, helminths, and acanthocephalans. Helminth groups identified were trematodes, cestodes and nematodes, where the nematodes were the most common GI parasites among them. A total of 20 genera types of GI parasites were explicitly identified; (i) five types of protozoan cysts; *Balantidium*, *Endolimax*, *Isospora*, *Entamoeba*,and an unidentified protozoan; (ii) two types of unidentified trematode ova; (iii) four types of cestode ova; *Hymenolepis*, *Bertiella*, *Diphyllobothrium*, and an unidentified cestode; (iv) eight types of nematode ova; *Strongyloides*, *Trichuris*, *Trichostrongylus*, *Oesophagostomum, Ascaris*, *Enterobius*, *Strongyle*/hookworm types and an unidentified nematode type; and (v) a single type of acanthocephalan ova; *Moniliformis* (Fig. 3).

### 3.2 Overall parasite prevalence

Out of 180 samples of macaque fecal samples examined, 62.2% (112/180) were infected with at least a single GI parasite type. Among the samples, 62.2% (112/180) were infected with helminths while protozoan infections were detected in only 8.3% (15/180). There was no significant difference in the overall prevalence of GI parasites among *M*. *s*. *aurifrons* (56.7% [34/60]), *M. s. sinica* (68.3% [41/60]) and *M*. *s*. *opisthomelas* (61.7% [37/60]) (χ^2^ = 1.749, *p* = 0.471).

However, the overall GI parasite prevalence significantly differed in the three population types representing each macaque subspecies. The highest significant prevalence was found in macaques that inhabit urban habitats (76.7% [46/60]) followed by macaques in suburban habitats (68.3% [41/60]), with the lowest prevalence reported from wild habitats (χ^2^ = 17.064, p < 0.05). Overall, macaques in urban (χ^2^ = 15.211, p < 0.05) and sub urban (χ^2^ = 8.620, p < 0.05) habitats had the highest significant prevalence in contrast to their wild counterparts (Table 1).

**Table 1:** Overall parasite prevalence and prevalence of multiple infections of GI parasites in urban, suburban, and wild macaque (*Macaca sinica*) populations.

Population type (n)	Overall	Prevalence of multiple infections % (n)				
	parasite prevalence	Parasite free	Single infection	Two infections	Three infections	Four infections
**Urban**						
Overall urban (60)	76.7 (46)^a^	23.3 (14)	38.3 (23)	26.7 (16)^e^	5.0 (3)	6.7 (4)
*M.s. aurifrons* (20)	70.0 (14)	30.0 (6)	30.0 (6)	35.0 (7)	5.0 (1)	–
*M.s. opisthomelas* (20)	70.0 (14)	30.0 (6)	40.0 (8)	20.0 (4)	5.0 (1)	5.0 (1)
*M.s. sinica* (20)	90.0 (18)^d^	10.0 (2)	45.0 (9)^f^	25.0 (5)	5.0 (1)	15.0 (3)
**Suburban**						
Overall suburban (60)	68.3 (41)^b^	31.7 (19)	35.0 (21)	21.7 (13)	8.3 (5)	3.3 (2)
*M.s. aurifrons* (20)	60.0 (12)	40.0 (8)	35.0 (7)	5.0 (3)	5.0 (1)	5.0 (1)
*M.s. opisthomelas* (20)	75.0 (15)^c^	25.0 (5)	35.0 (7)	20.0 (4)	5.0 (3)	5.0 (1)
*M.s. sinica* (20)	70.0 (14)	30.0 (6)	35.0 (7)	30.0 (6)	5.0 (1)	–
**Wild**						
Overall wild (60)	41.7 (25)	54.3 (35)	26.7 (16)	10.0 (6)	5.0 (3)	–
*M.s. aurifrons* (20)	40.0 (8)	60.0 (12)	35.0 (7)	5.0 (1)	–	–
*M.s. opisthomelas* (20)	40.0 (8)	60.0 (12)	30.0 (6)	5.0 (1)	5.0 (1)	–
*M.s. sinica* (20)	45.0 (9)	55.0 (11)	5.0 (3)	20.0 (4)	3.3 (2)	–

Significant difference between overall parasite prevalence in.

single parasite types in urban and wild populations of *M*. *s*. *sinica* (χ 2 = 4.286, p = 0.038).

Two types of parasites in urban and wild populations of *M*. *s*. *aurifrons* (χ 2 = 5.625, p = 0.017).

a Urban and wild populations (χ 2 = 15.211, p < 0.05).

b Suburban and wild populations (χ 2 = 8.620, p < 0.05).

c Sub urban and wild populations of *M*. *s*. *opisthomelas* (χ 2 = 5.013, p = 0.025).

d Urban and wild populations of *M*. *s*. *sinica* (χ 2 = 9.231, p = 0.002) Significant difference between macaques having infections with.

e Two types of parasites in overall urban and overall wild populations (χ 2 = 5.566, p = 0.018).

### 3.3 Mixed infections

Infected macaques harbored multiple parasitic infections, with numbers that ranged between one and four per individual. Of the overall number of macaques in the three population types, i.e., urban, suburban, and wild, most animals were infected with a single type of parasite, *i.e.,* urban (38.3% [23/60]), suburban (35% [21/60]) and wild, (26.7% [16/60]). Collectively, macaques having infection with two types of parasites in urban (26.7% [16/60]) were significantly higher when compared to the wild (10% [6/60]) (χ^2^ = 5.566, p = 0.018) but there was no significant difference between sub urban (21.7% [13/60]) and wild (χ^2^ = 3.064, p = 0.08) populations. Conversely, no significant difference was recorded among the percentage of toque macaques harbouring three parasite types in urban (5% [3/60]), sub urban (8.3% [5/60]) and wild (5% [3/60]) habitats. The maximum score for multiple infections per single macaque was four types of parasites per individual recorded in urban (6.7% [4/60]) and suburban (3.3% [2/60]) populations. Conversely, the highest number of parasite types per infected macaque in the wild population, numbered three (Table 1).

With respect to single parasitic infections in each macaque sub species, only *M. s. sinica* in the urban habitat showed a significant difference compared to their wild counterparts (χ 2 = 4.286, p = 0.038). Conversely, only urban population of *M*. *s*. *aurifrons* indicated a significant difference compared to their wild population, harbouring two types of parasitic infections in each macaque (χ 2 = 5.625, p = 0.017) (Table 1).

### 3.4 Parasite prevalence and species richness of the three-macaque subspecies inhabiting diverse habitat types

**Fig. 4:**
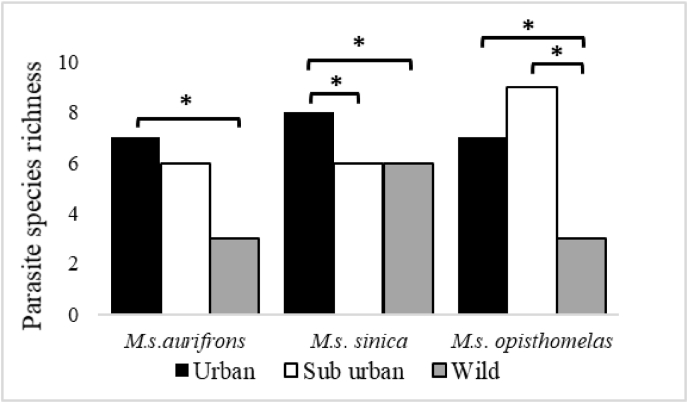
Number of parasite genera types (species richness) infecting *M. s. aurifrons, M. s. sinica and M. s. opisthomelas* in urban, suburban, and wild habitats.

Overall prevalence of infection did not differ among *M*. *s*, *s*. *aurifrons* in urban (70%), suburban (60%) and wild (40%) populations (χ^2^ = 3.801, p = 0.15), or for any of the GI parasite genera types documented (Table 1). Of *M*. *s*. *aurifrons* populations in the three habitat types s, *Strongyloides* type was the most frequent nematode infection, followed by *Strongyle*/hookworm type and *Trichuris* type while *Balantidium* type was the commonest amongst protozoans. *M*. *s*. *aurifrons* from the urban population were exclusively infected with *Isospora* type and *Trichostrongylus* type*.* Infection by *Diphyllobothrium* type and *Ascaris* type were confined to suburban animals, while *Enterobius*

type was recorded in macaques inhabiting their natural habitat (Table 2). A significant difference existed between the total number of parasite genera types infecting *M*. *s*. *aurifrons* urban (n = 7), suburban(n = 6) and wild (n = 3) (p = 0.045) habitats. Urban living *M*. *s*. *aurifrons* population harbored more parasite species than those living in the wild (Z = −2.484, p = 0.013) (Fig. 4).

**Table 2:** Prevalence (%) of GI parasite infections in *Macaca sinica aurifrons, M. s. sinica and M. s. opisthomelas* in urban, suburban, and wild habitats.

Host	Prevalence of GI parasite type% (n) (N = 180)								
Habitat type	*M.s. aurifrons* (n = 60) (n = 20 per habitat)			*M.s. sinica* (n = 60) (n = 20 per habitat)			*M.s. opisthomelas* (n = 60) (n = 20 per habitat)		
Parasite genera types	U	SU	W	U	SU	W	U	SU	W
**Protozoan types**									
*Balantidium*	20(4)	15(3)	–	–	–	–	5(1)	10(2)	5(1)
*Isospora*	5(1)	–	–	–	–	–	–	5(1)	–
*Entamoeba*	–	–	–	–	–	–	–	5(1)	–
*Endolimax*	–	–	–	5(1)	–	5(1)	–	–	–
Unidentified protozoa	–	–	–	–	–	–	–	5(1)	–
Total protozoan infection	25(5)	15(3)	–	5(1)	–	5(1)	5(1)	20(4)	5(1)
**Trematode types**									
Unidentified trematodes	–	–	–	–	5(1)	–	5(1)	–	–
**Cestode types**									
*Diphyllobothrium*	–	5(1)	–	–	–	–	–	–	–
*Bertiella*	–	–	–	–	5(1)	–	–	–	–
*Hymenolepis*	–	–	–	–	–	25(5)	–	–	–
Unidentified cestode	–	5(1)	–	5(1)	–	–	–	–	–
**Nematode types**									
*Strongyloides*	45(9)	60(12)	35(7)	85(17)	70(14)	35(7)	60(12)	45(9)	30(6)
*Trichuris*	15(3)	–	10(2)	25(5)	10(2)	15(3)	5(1)	20(4)	–
*Ascaris*	–	5(1)	–	15(3)	15(3)	–	10(2)	10(2)	–
*Strongyle*/Hook worm	20(4)	10(2)	–	15(3)	5(1)	–	25(5)	35(7)	20(4)
*Trichostrongylus*	20(4)	–	–	10(2)	–	–	–	–	–
*Oesophagostomum*	–	–	–	5(1)	–	–	5(1)	5(1)	–
*Enterobius*	–	–	5(1)	–	–	5(1)	–	–	–
Unidentified nematode	5(1)	–	–	–	–	–	–	–	–
Total helminths infection	70(14)	60(12)	40(8)	90(18)	70(14)	45(9)	70(14)	75(15)	40(8)
**Acanthocephalan type**									
*Moniliformis*	–	–	–	5(1)	–	–	–	–	–

U, urban; S.U, suburban; W, wild.

There was a significant difference in the overall prevalence of parasites among the urban (70%), suburban (75%) and wild (40%) populations of *M*. *s*. *opisthomelas* (χ^2^ = 6.063, p = 0.048). However, when comparisons were drawn among populations, macaques in the suburban population (75%) had the highest significant prevalence of parasites than in the wild (40%) (χ^2^ = 5.013, p = 0.025), while there was no significant difference between wild and urban living macaques (χ^2^ = 3.636, p = 0.057) (Table 1).

*M*. *s*. *opisthomelas* was exceptional among the three-macaque subspecies, as prevalence of specific parasite types among the urban, suburban, and wild populations did not differ. Infection by *Isospora* type and *Entamoeba* type were confined to macaques in the suburban habitat (Table 2). There was a significant difference between the total number of parasite genera types infecting *M*. *s*. *opisthomelas* in urban (n = 7), suburban (n = 9) and wild (n = 3) (p = 0.028) habitats. Comparison of parasite richness in the different habitat types, was high in both suburban (n = 9) (Z = −2.022, p = 0.043) and urban populations (n = 7) (Z = −2.514, p = 0.012) compared to the wild (n = 3) (Fig. 4).

The overall prevalence of infection significantly differed among *M*. *s*. *sinica* urban (90%), suburban (70%), and wild (45%) populations (χ^2^ = 9.397, p = 0.009). Parasite prevalence in the urban macaque population was significantly higher than of the wild (χ^2^ = 9.231, p = 0.002) (Table 1). There was no significant difference in the relative prevalence of individual parasite types except for *Strongyloides* type in urban and wild populations (χ^2^ = 10.417, p = 0.001). Comparable to the other two subspecies*, Strongyloides* type was the frequently recorded nematode infection followed by *Trichuris* type, in *M*. *s*. *sinica* in all three habitat types*.* It is noteworthy that *Hymenolepis* type*, Bertiella* type, and *Moniliformis* type were confined to *M*. *s*. *sinica* among the three subspecies of endemic macaques (Table 2). Overall parasite species high in the urban macaques (n = 8) compared to their suburban and wild counterparts (n = 6) (Z = −1.017, p = 0.02) (Fig. 4).

### 3.5 Parasite ova/cyst per gram (OPG/CPG) feces

The index OPG/CPG indicates the parasite load in a host. In *M*. *s*. *aurifrons* the highest OPG was of *Strongyloides* type (OPG = urban; 3.33 ± 1.42, suburban; 1.72 ± 1.14, wild; 1.00 ± 0.51), though the maximum CPG was recorded for *Balantidium* type in both urban and suburban populations. All other analyzed OPG and CPG were relatively low. No significant variation among individual OPG was recorded except for the EPG of *Strongyloides* type (Table 3). The urban population of *M. s. aurifrons* manifested a significantly higher OPG of *Strongyloides* type than those from the wild (Z = -2.036, p = 0.042) population.

**Table 3:** Mean parasite ova/cyst per gram (OPG/CPG) feces of different parasite types infecting *Macaca sinica aurifrons, M.s. sinica and M.s. opisthomelas* in urban, suburban and wild habitats.

Host	Mean parasite OPG/CPG ± (SD)								
Habitat type	*M.s. aurifrons*			*M.s. sinica*			*M.s. opisthomelas*		
Parasite genera types	U	SU	W	U	SU	W	U	SU	W
**Protozoan types**									
*Balantidium*	0.67±	0.89±	–	–	–	–	0.67^a^	0.83±	0.67^a^
	(0.27)	(0.38)	–	–	–	–	–	(0.71)	–
*Isospora*	1.0^a^	–	–	–	–	–	–	0.33^a^	–
*Entamoeba*	–	–	–	1.00^a^	–	1.00^a^ -	–	0.67^a^	–
*Endolimax*	–	–	–	–	–	–	–	–	–
Unidentified protozoa	–	–	–	–	–	–	–	0.33^a^	–
**Trematode types**									
Unidentified Trematodes	–	–	–	–	0.33^a^	–	0.33^a^	–	–
**Cestode types**									
*Diphyllobothrium*	–	1.33^a^	–	–	–	–	–	–	–
*Bertiella*	–	–	–	–	0.33^a^	–	–	–	–
*Hymenolepis*	–	–	–		–	2.53± (1.42)	–	–	–
Unidentified cestode		2.00^a^		1.00^a^					
**Nematode types**									
*Strongyloides*	3.33±	1.72±	1.00±	4.35±	5.14±	1.14±	4.83±	1.41±	0.67±
	(1.42)	(1.14)	(0.51)	(5.33)	(5.81)	(0.77)	(7.67)	(0.96)	(0.37)
*Trichuris*	1.00±	–	0.33±	1.00±	1.17±	1.33±	1.00^a^	1.25±	–
	(0.33)		(0)	(0.71)	(1.18)	(0.88)		(0.69)	–
*Ascaris*		1.67^a^	–	0.33± (0)	1.33±	–	0.67±(0.47)	0.50±(0.24)	0.83±
	–		–		(1.21)	–			(0.43)
*Strongyle*/Hook worm	1.50±	2.00±(0.47)	–	4.11± (2.41)	4.33^a^	–	2.07± (1.38)	1.14± (0.43)	–
	(0.88)	–	0.67^a^	0.33±	–	1.00^a^	–		–
*Oesophagostomum*	1.50±	–	–	(0)	–	–	1.67^a^		–
	(0.69)	–	–	1.33^a^	–	–	–	–	–
*Enterobius*	–	–		–	–		–	1.33^a^	–
Unidentified nematode	–	–		–	–		–	–	
**Acanthocephalan type**									
*Moniliformis*	0.33^a^			1.33^a^	–			–	
	–							–	

U-urban; S.U.- suburban; W- wild.

a Infections were confined to a single individual macaque.

The highest OPG for *M*. *s*. *opisthomelas* was recorded for *Strongyloides* type from urban and suburban habitats (OPG = urban; 4.83 ± 7.67, suburban; 1.41 ± 0.96). The highest protozoan CPG was of *Balantidium* type recorded in the suburban population (0.83 ± 0.71). OPG of *Strongyloides* type differed among urban and wild populations (Z = -2.167, p = 0.03). No other significant comparisons were recorded among the individual parasite genus types in *M*. *s*. *opisthomelas* inhabiting the three examined habitats (Table 3).

Compared to the wild population, OPG of individual parasite types differed for *M*. *s*. *sinica* among both urban (Z = -3.625, p < 0.05) and sub urban (Z = -2.636, p = 0.008) populations. Nevertheless, the highest OPG of *Strongyloides* type was recorded from suburban *M*. *s*. *sinica* samples (5.14 ± 5.81) than those from the other two macaque subspecies. The maximum CPG was of *Endolimax* type (1.00) which was recorded in a single individual macaque each from urban and wild populations.

## 4 Discussion

It is well established that demographic and anthropogenic changes exert influence on the emergence of zoonotic diseases (Jones et al., 2008; Lane et al., 2011). Hitherto, GI parasite infections in toque macaques, especially with the influence of urban landscape on the wildlife – pathogen relationship received less emphasis. This is the first study of its kind, based on the impact of urbanization on GI parasites in the three endemic subspecies of toque macaques in Sri Lanka. As hypothesized, *M*. *s*. *opisthomelas, M*. *s*. *aurifrons* and *M. s. sinica* in urbanized habitats harboured a higher prevalence and parasite richness of GI parasites in contrast to their wild counterparts. It was observed that there is a marked difference in daily activity, ranging patterns, food, and water sources of macaque troops in urban settings, compared to those in the wild. These observations reiterate several studies on different macaque species and other primates, globally; these reports are indicative of alterations in population structure of parasite infections in primates with the level of anthropogenic interactions and environment modifications (Sleeman et al., 2000; Gillespie et al., 2005; Ekanayake et al., 2006; Wenz et al., 2010; Lane et al., 2011; Hodder and Chapman, 2012; Hussain et al., 2013; Wenz-mücke et al., 2013). Furthermore, climatic factors and altitude also can be considered as factors that influence the survival and infection of parasites in free-ranging macaques. Among the three sub species inhabiting different climatic zones of the island, *M. s. sinica* found in the dry zone harbour a higher prevalence of GI parasites compared to the other two subspecies. This may be due to increase infection success and faster development in parasites in the high temperature than in relatively low temperature ranges (Labaude et al., 2020). Maximum temperature is an important variable in determining soil-transmitted helminth distribution owing to effect of heat and low humidity of the soil on the embryonation, development and survival of free-living infective stages (Brooker et al., 2003).

Overall, 20 parasite genera types were identified in the GI parasite survey that contributed to the parasite diversity of toque macaques; the *Strongyloides* type was responsible for the highest prevalence and parasite load (OPG) in all three subspecies of hosts followed by *Strongyle/*hookworm and *Trichuris* (whipworm) types, respectively. Infection by *Strongyloides* occurs by accidental oral mucosa or skin penetration of third-stage filariform larvae in the soil (Grove, 1996). Even though macaques are primarily arboreal (Nekaris and de Silva Wijeyeratne, 2009), during the observation period from 8.00 to 16.00 h, both urban and suburban troops dwelled on the ground for about 80% of the time, while this was limited to an average of 15% by the toque groups in the wild (authors’ personal observations). Therefore, predominant ground living behaviour and close association of individuals within the macaque group may be the major reason for the high prevalence of *Strongyloides* infection (Altmann and Muruthi, 1988; Ekanayake et al., 2006; Griffin and Nunn, 2012). Strongyloidiasis in humans is described as a potential zoonotic disease (Nutman, 2016). Pathological effects of this infection in primates vary from vomiting, diarrhea, and severe dehydration (Strait et al., 2012) while fibrosis of the intestine results in humans (Mukerjee et al., 2003). In severe infection, macaques may cope with similar clinical problems as humans, and several fatal cases have been reported in the gibbon, orangutan, and woolly monkeys (Pillers and Southwell, 1929; DePaoli and Johnsen, 1978; Uemura et al., 1979) elsewhere in the world.

*Strongyle/*hookworm type infection, the second-highest GI parasite prevalence was recorded in all three-macaque subspecies inhabiting urbanized habitats. However, in the wild, this infection was restricted to *M*. *s*. *opisthomelas.* Individual primates with more grooming partners can have a significantly higher infection of hookworms (Wren et al., 2016) and grooming related with the physiological stress levels in macaques (Shutt et al., 2007). Therefore, when individuals in the population are more stressed and defense mechanisms fail (Mul et al., 2007) it could lead to greater susceptibility to infections in such individuals than in others. It eventually poses a threat to macaque populations as this infection causes tissue damage, retarded growth, anemia, and inflammation (Hussain et al., 2013; Seguel and Gottdenker, 2017). More importantly, macaques in urbanized habitats create human-macaque interfaces that disturb the dynamics of hookworm infection and infected macaques can be a threat to closely interacting humans (Seguel and Gottdenker, 2017).

*Trichuris* (whipworm) infection was reported in all three-macaque subspecies in habitats except for M. *s. opisthomelas* wild populations (in montane regions)*,* and suburban populations of *M*. *s*. *aurifrons* in the wet zone*.* This reiterates alteration of parasite patterns due to geographic and climatic conditions (Sharma et al., 2013). In previous studies, *Trichuris* infection in Asian primates was found to be less common, and macaques may acquire it because of the close association with grey langurs (Dewit et al., 1991). This may explain the relatively high prevalence of *Trichuris* type in *M. s. sinica* in this study, as their troops were mostly observed foraging together with grey langurs. *Trichuris* infection occurs orally, and ova can survive for a longer period outside the host, in severe environmental conditions. Severe whipworm infections can produce clinical signs such as anorexia, diarrhea resulting sometimes in death (Le et al., 2020).

Most of the suburban and a single urban population of macaques were recorded in places of religious worship, where local pilgrims and tourists visited throughout the year. Consequently, the presence of infected macaques with their randomly dispersed feces found in those areas may pose a threat to public health, as in those religious and archeological sites, people usually walk barefooted. This may increase the possibility of humans to acquire soil-transmitted helminth infections.

Nematode infections pose high potential for zoonotic transmission with their direct and simple life cycles while the infections by cestodes and acanthocephalan are normally rare and only causes diarrhea and abdominal pain in primates (Torgerson and Macpherson, 2011; Strait et al., 2012). Even though *Bertiella and Moniliformis* infections were restricted to individual M. *s. sinica* macaques, it may indicate the possible transmission of these parasites from other animals such as rodents and mites to macaques (Elliott, 2007; Kowalewski et al., 2017).

Confirming previous studies, the prevalence of protozoans was lower compared to the helminth infections in macaques in the current study (Kouassi et al., 2015; Adhikari and Dhakal, 2018; Adrus et al., 2018; Kumar et al., 2018; Zhang et al., 2019). The contrary was reported by Li et al. (2017) with 40.1% of macaques screening positive for protozoan and 29.6% for helminth infections. Kurniawati et al. (2020) reiterated protozoan was the most prevalent infection (89%) compared to helminth infections (66%) in long-tailed macaques. All five types of protozoans detected in the current study, were of relatively high prevalence; *Balantidium* type > *Isospora* type in both *M*. *s*. *aurifrons* and *M*. *s*. *opisthomelas*. All recorded protozoan's manifest zoonotic transmission and are human pathogens except for *Isospora* type which is a common parasite of cats and dogs (Youn, 2009; Ferreira et al., 2011). In general, protozoan infections are highly prevalent due to the capability of cysts to survive even in harsh environments, and their one host-life cycle. These recorded protozoans can transmit directly through the fecal-oral route via contaminated food or water sources (Osman et al., 2016). *Balantidium* sp. is known as the only ciliated protozoan that commonly infects humans and several animals. It can be pathogenic to both macaques and humans by causing clinical symptoms of Balantidiasis which include diarrhea and dysentery (Hussain et al., 2013). During the current study macaque troops were observed using the same water source for drinking as well as for bathing which can be the major reason that facilitates transmission of protozoan infections. Ekanayake et al. (2006) observed that wild primates using land areas heavily soiled by human and livestock feces, harbored more parasitic protozoans common in humans, than their wild counterparts. More importantly, *Balantidium* infection in lactating female macaques can reduce the fat of milk and inhibit the development of lactating mothers by reducing activity levels and impairing their foraging behaviour (Hinde, 2007). Importantly, it can compromise the growth of infants which can cause several health implications to macaque populations.

Several studies conducted in both children and adults in Sri Lanka from 1924 to 2019, found that school children across the country were positive for hookworm, whipworm, roundworm and *Bertiella* infection, especially those living in urban areas (Amarasinghe et al., 2020; De Silva et al., 1994; Sorensen et al., 1996; Gunawardena et al., 2011; Galgamuwa et al., 2016; Ediriweera et al., 2019)., The reason attributed to *Bertiella* infections was that the main reservoir hosts, the toque macaques, were regular visitors to human settlements (Amarasinghe et al., 2020), which may pose a threat, especially to small children as well as to domestic animals. Furthermore, alternations to the availability of foraging and roosting sites and the quality and quantity of food macaques consumed due to anthropogenic activities and urbanization, may lead in several ways to increase the parasite burden in macaque populations (Weyher et al., 2006). Importantly, crowding in a fragmented area with small space, frequent locomotion on the ground, grooming behaviour and repeated use of the same area may not only increase the possibility of parasite transmission but also, increase chances to re-infect within the troop members. Although there are no fatality reports on diseases caused by GI parasites in macaques and humans in Sri Lanka, it is prudent to take precautionary measures. Therefore, it is important to encourage creating more natural habitats suitable for macaques within urban areas, the introduction of proper waste disposal methods, avoid feeding macaques, continuous deworming practices and health education, to reduce health impacts on both humans and co-existing macaque populations.

Several novel host-parasite associations were established through the current study; this is the first report of *Moniliformis* type infection in macaques globally and the first report of *Isospora* type, *Trichostrongylus* type*, Enterobius* type and *Diphyllobothrium* type infection in *M*.*s*.*aurifrons* (Huffman et al., 2013; Thilakarathne et al., 2021), *Endolimax* type*, Moniliformis* type and *Bertiella* type infection in *M*.*s*.*sinica*(Dewit et al., 1991; Ekanayake et al., 2006; Huffman et al., 2013; Thilakarathne et al., 2021) and *Isospora* type, *Balantidium* type, *Strongyloides* type, *Ascaris* type and *Oesophagostomum* type infections in *M. s. opisthomelas* (Huffman et al., 2013).

In summation, results from this study lead to two conclusions; Firstly, macaques that live in urban and suburban areas, closer to human settlements harboured increased prevalence and parasite richness compared to wild macaques in their natural habitat. Secondly, infections by soil-transmitted parasites in macaques were relatively high. As two of the subspecies of macaques (*M*. *s*. *aurifrons* and *M*. *s*. *sinica*) are endangered and the other (*M*. *s*. *opisthomelas*) is critically endangered, further studies are required to gauge whether the higher prevalence of parasites encountered may pose a threat to their population abundance and daily activity patterns. Further, identification of recorded parasites up to the species level is also important in assessing major health risks of these parasitic infections. Almost all the parasites identified in the current study are of zoonotic potential. Given the genetic relatedness of humans and macaques, disease transmission between them may be potent. Therefore, to verify the existent risk, it is necessary to prove whether humans who share the same environment are indeed infected with any diseases caused by zoonotic GI parasites found in co-existing macaque populations. Such future efforts will help prevent transmission of zoonotic parasites, which is an important implication for primate conservation efforts while ensuring public health and safety.

## Declaration of interests

The authors declare that they have no known competing financial interests or personal relationships that could have appeared to influence the work reported in this paper.
